# Prevalence and antibiotic resistance of bacterial pathogens isolated from childhood diarrhea in Beijing, China (2010–2014)

**DOI:** 10.1186/s13099-016-0116-2

**Published:** 2016-06-13

**Authors:** Mei Qu, Bing Lv, Xin Zhang, Hanqiu Yan, Ying Huang, Haikun Qian, Bo Pang, Lei Jia, Biao Kan, Quanyi Wang

**Affiliations:** Department of Infectious and Endemic Diseases Control, Beijing Key Laboratory of Diagnostic and Traceability Technologies for Food Poisoning, Beijing Center for Disease Prevention and Control, No. 16 He Ping Li Middle Street, Beijing, 100013 People’s Republic of China; State Key Laboratory for Infectious Disease Prevention and Control, National Institute for Communicable Disease Control and Prevention, Changping, Beijing, 102206 People’s Republic of China

**Keywords:** Diarrhea surveillance, Diarrheagenic bacteria, Antimicrobial resistance, Children

## Abstract

**Background:**

Diarrhea is one of the main causes of morbidity and mortality among children less than 5 years of age worldwide, and its causes vary by region. This study aimed to determine the etiologic spectrum, prevalent characteristics and antimicrobial resistance patterns of common enteropathogenic bacteria from diarrheagenic children in Beijing, the capital of China.

**Methods:**

Stool samples were collected from 2524 outpatients who were aged 0–5 years in Beijing, China during 2010–2014. Microbiological methods, real-time PCR and antimicrobial susceptibility test were used to identify the bacterial causes and antimicrobial resistance patterns in the isolates.

**Results:**

Of the 2524 patients screened, we identified the causes of 269 cases (10.7 %) as follows: diarrheagenic *Escherichia coli* (4.6 %), *Salmonella* (4.3 %), *Shigella* (1.4 %) and *Vibrio parahaemolyticus* (0.4 %). Atypical EPEC, *Salmonella enteritidis*, *Shigella sonnei* and serotype O3:K6 were the most common serogroups or serotypes of the four etiological bacteria. The prevalence of pathogens was correlated with age, season and clinical symptoms. The highest proportion of all causative bacteria was found in children aged 3–5 years and in summer. The clinical symptoms associated with specific bacterial infection, such as fever; abdominal pain; vomiting; and watery, mucus, and bloody stool, were observed frequently in diarrheal patients. *Salmonella* showed moderate rates of resistance (40–60 %) to ampicillin, nalidixic acid, streptomycin and sulfisoxazole. Resistance to at least three antimicrobials was found in 50 % of isolates. Of the top three serotypes in *Salmonella*, high-level antimicrobial resistance to single and multiple antibiotics was more common among *Salmonella typhimurium* and *Salmonella* 1, 4, [5], 12:i:- than among *S. enteritidis.* More than 90 % of *Shigella* isolates showed more alarming resistance to most antibiotics, with a widened spectrum compared to *Salmonella*.

**Conclusion:**

Constant antibiotic surveillance is warranted because the bacteria were highly resistant to various antimicrobials. Our study contributes to the strengthening of the existing surveillance system and provides aid for effective prevention and control strategies for childhood diarrhea.

**Electronic supplementary material:**

The online version of this article (doi:10.1186/s13099-016-0116-2) contains supplementary material, which is available to authorized users.

## Background

Diarrheal disease continues to be one of the foremost public health issues worldwide, with more than 1.5 million deaths each year, mostly in children under 5 years of age in industrializing countries. The average diarrheal disease incidence is an average of 2–3 episodes per child per year [1, 2]. In China, approximately 40,000 children suffer from diarrhea annually, especially during the first 2 years of life, and China remains one of the highest incidence countries [3]. Although diarrhea-associated mortality has roughly halved in the past 20 years, there is no indication that disease morbidity has decreased [4]. Moreover, there has been an alarming increase in the rate of resistance to commonly used antimicrobials. Therefore, acute diarrhea represents an important childhood ailment requiring treatment and prevention in China.

Recently, several specific diarrhea surveillance projects were conducted to explore the prevalence and etiology of bacterial infectious diseases in different regions in China [5–7]. Starting in April 2010, we established a real-time web-based diarrheal disease surveillance system based on sentinel hospitals for an unselected population of outpatients, which focused on common enteropathogenic bacteria accompanying clinical features, epidemiological information and laboratory confirmation in Beijing, China [8]. Through 5-year surveillance efforts and data accumulation, we aimed to identify the prevalence of the etiological spectrum, temporal distribution and antibiotic resistance patterns of bacterial pathogens associated with childhood diarrhea.

## Results and discussion

### Demography of enrolled subjects

From April 2010 to December 2014, 2524 stool specimens from outpatient children aged 0–5 years with acute diarrhea were collected and tested for bacterial pathogens. The outpatients comprised 1616 males and 908 females (male:female ratio = 1.8:1 and mean age 1.2 ± 1.1 years). Male children had a higher diarrheal incidence than female children, which was similar to the findings of other studies, despite the unknown explanation [9–11]. The age distribution showed that more than 90 % of children suffering from diarrhea were less than 2 years old (n = 2341, 92.7 %), and prevalence progressively decreased as age increased, in agreement with previous studies [11, 12]. This difference in prevalence may be due to the gradual reduction in protective antibodies from the mother before the children have developed mature nervous and immune response against various infections [13]. Childhood acute diarrhea was more frequent in the summer than in other seasons. Of the 2524 enrolled subjects with an average frequency of diarrhea of 4.9 ± 2.4, 68.3 % presented with mucus stool, 30.0 % with watery stool and 2.5 % with bloody stool. The other clinical symptoms were not obvious, such as fever, accounting for 7.2 % of the total, vomiting (6.5 %) and abdominal pain (4.4 %). However, 20.1 % of the diarrheal children had a history of antibiotic use prior to admission (Table 1).

**Table 1:** Demographic and illness characteristics of bacterial pathogens associated with childhood diarrhea in Beijing

Characteristic	All enrolled subjects (n = 2524)	*Diarrheagenic E. coli* (n = 117)^a^	*Salmonella* spp. (n = 109)	*Shigella* spp. (n = 37)	*V. parahaemolyticus* (n = 10)	Total of all pathogens (n = 269)
Age (years), n (%)						
<1	1497 (59.3)	53 (3.5)	44 (2.9)	5 (0.3)	6 (0.4)	107 (7.1)
1–2	844 (33.4)	58 (6.9)	52 (6.2)	16 (1.9)	4 (0.5)	127 15.0)
3–4	131 (5.2)	5 (3.8)	11 (8.4)	9 (6.9)	0 (0.0)	25 (19.1)
=5	52 (2.1)	1 (1.9)	2 (3.8)	7 (13.5)	0 (0.0)	10 (19.2)
Mean age ± SD	1.2 (1.1)	1.3 (1.0)	1.5 (1.1)	2.9 (1.8)	1.1 (0.7)	1.6 (1.3)
*P* value	–	0.002	<0.001	<0.001	0.836	<0.001
Sex, n (%)						
Male	1616 (64.0)	70 (4.3)	71 (4.4)	26 (1.6)	3 (0.2)	170 (10.5)
Female	908 (36.0)	47 (5.2)	38 (4.2)	11 (1.2)	7 (0.8)	99 (10.9)
*P* value	–	0.333	0.517	0.302	0.025	0.765
Seasons, n (%)						
Spring (Mar–May)	584 (23.1)	22 (3.8)	28 (4.8)	0 (0.0)	1 (0.1)	50 (8.6)
Summer (Jun–Aug)	1029 (40.8)	62 (6.0)	50 (4.9)	31 (3.0)	6 (0.6)	146 (14.2)
Autumn (Sep–Nov)	615 (24.4)	27 (4.4)	25 (4.1)	6 (0.9)	3 (0.5)	61 (9.9)
Winter (Dec–Feb)	296 (11.7)	6 (5.1)	6 (5.1)	0 (0.0)	0 (0.0)	12 (2.0)
*P* value	–	0.017	0.229	<0.001	0.397	<0.001
Symptom, n (%)						
Fever (>38 °C)	181 (7.2)	3 (2.6)^b^	21 (19.3)^b^	14 (37.8)^b^	0 (0.0)	38 (14.1)^b^
Abdominal pain	112 (4.4)	4 (3.4)	8 (7.3)	7 (18.9)^b^	0 (0.0)	19 (7.1)^b^
Vomiting	165 (6.5)	6 (5.1)	5 (4.6)	9 (24.3)^b^	0 (0.0)	20 (7.4)
Dehydration	11 (0.4)	0 (0.0)	0 (0.0)	0 (0.0)	0 (0.0)	0 (0.0)
Watery diarrhea	758 (30.0)	38 (32.5)	20 (18.3)^b^	8 (21.6)	1 (10.0)	66 (24.5)^b^
Mucus diarrhea	1724 (68.3)	76 (65.0)	87 (79.8)^b^	26 (70.3)	8 (80.0)	193 (71.7)
Bloody diarrhea	63 (2.5)	2 (1.7)	7 (6.4)^b^	3 (8.1)^b^	1(10.0)	13 (4.8)^b^
Meandiarrhea frequency ± SD	4.9 (2.4)	4.3 (2.3)	5.7 (2.6)	6.9 (3.6)	4.1 (1.6)	5.2 (2.8)
Antibiotic consumption	507 (20.1)	25 (21.4)	28 (25.7)	23 (62.2)	5 (50.0)	79 (29.4)

^a^One specimen was identified by mixed infection of EPEC and EAEC, therefore n = 117; *SD* standard deviation

^b^Chi square test was calculated by each symptomdue to different pathogen, all significant *P* values below 0.05 was noted

### Distribution of pathogenic bacteria by species and serotype

At least one enteric pathogenic bacteria was isolated from 269 (10.7 %) of the 2524 stool specimens, with a total of 274 isolates. The prevalence of different bacteria is shown in Table 2. Diarrheagenic *Escherichia coli* (DEC) was the most common pathogen, isolated in 118 specimens (43.1 %, 118/274), followed by *Salmonella* in 109 (39.8 %), *Shigella* in 37 (13.5 %) and *Vibrio parahaemolyticus* in 10 (3.6 %). Among samples containing pathogenic bacteria (n = 269), single infections were observed in 264 (99.1 %) cases, and the remaining five cases of mixed infections had two pathogens, including DEC and *Salmonella* (n = 3), DEC and *Shigella* (n = 1), enteropathogenic *E. coli* (EPEC) and enteroaggregative *E. coli* (EAEC) (n = 1).

**Table 2:** Distribution of different enteropathogenic bacteria(n = 274) isolated from diarrheal patients

Any simple pathogen	No. of isolates (%)
Diarrheagenic *E.coli* (n = 118, 5 serogroups)	118 (43.1)
EPEC	
Atypical (eae+ , *bfp*A−)	58 (49.2)
Typical (eae+ , *bfp*A +)	9 (7.6)
EAEC	
*agg*R+	28 (23.7)
ETEC	
*est*+	9 (7.6)
*elt*+	6 (5.1)
Both+	2 (1.7)
EIEC (*ipa*H +)	5(4.2)
STEC (eae + , *stx*1 +)^a^	1 (0.8)
*Salmonella* spp. (n = 109, 33 serotypes)	109 (39.8)
*S. enteritidis*	35 (32.1)
*S. typhimurium*	15 (13.8)
*Salmonella* 1, 4, [5], 12:i:-	13 (11.9)
*S. agona*	10 (9.2)
*S. paratyphi C*	4 (3.7)
*S. infantis*	3 (2.8)
*S. braenderup*	2 (1.8)
*S. tennessee*	2 (1.8)
Other serotypes^b^	25 (22.5)
*Shigella* spp. (n = 37, 4 serotypes)	37 (13.5)
*S. sonnei*	32 (86.5)
*S. flexneri* 2a	2 (5.4)
*S. flexneri* 4c	2 (5.4)
*S. dysenteriae* 2	1 (2.7)
*V. parahaemolyticus* (n = 10, 3 serotypes)	10 (3.6)
*V. parahaemolyticus* O3:K6	8 (80)
*V. parahaemolyticus* O3:KUT	1 (10)
*V. parahaemolyticu*s O1:KUT	1 (10)
Total	274 (100)
Mixed infections (n = 5)	
EPEC/*Salmonella* (*S. enteritidis*)	1
EPEC/*Salmonella* (*S. infantis)*	1
EAEC/*Salmonella* (*S. typhimurium*)	1
EPEC/*Shigella* (*S. sonnei*)	1
EPEC/EAEC	1

^a^The serotype of STEC is O26:K60

^b^The sum of the other serotypes excluding the top 8 serotypeslisted above

The prevalence pattern of serogroup and serotype by region is diverse in different studies. DEC is an important etiologic agent of diarrhea in developing countries [14]. In this study, 118 DEC isolates were detected from nine virulence genes. The most prevalent category was EPEC, followed by EAEC, enterotoxigenic *E. coli* (ETEC), enteroinvasive *E. coli* (EIEC), and Shiga toxin-producing *E. coli* (STEC). Among the 67 EPEC strains, 58 strains carrying the *eae* gene only were grouped as atypical EPEC, and the other nine strains harboring the *bfp*A and *eae* genes were typical EPEC. The atypical EPEC were more prevalent than typical EPEC (49.2 vs. 7.6 %), which is an emerging trend in developing and developed countries [15, 16]. Although the etiologic role of this sub-pathotype of EPEC in diarrhea has not been definitively established [17], this finding underscores the emergence of atypical EPEC worldwide. EAEC (23.7 %) was the second most abundant *E. coli*, although it was the most prevalent in previous studies [7, 12, 18, 19]. This observed rate is in sharp contrast with the reported low and high detection rates of 17.4 and 31.8 % in two previous studies from China [6, 7]. In the 17 ETEC strains, the *est* and *elt* genes were detected in 9 and 6 strains, respectively, and both *est* and *elt* positive were found in two strains. Five EIEC strains produced *ipa*H gene, and the only STEC isolate positive for *stx*1 and *eae* was O26:K60. The low prevalence of STEC and EIEC strains in this study is in agreement with the previous studies in China [6, 7] but differs from those in Iran and Tunisia, with high isolation rates [12, 20]. As the four most prevalent serovars of *Salmonella* isolates, *Salmonella enteritidis*, *Salmonellatyphimurium*, *Salmonella* 1, 4, [5], 12:i:- and *Salmonellaagona* represented 32.1, 13.8, 11.9 and 9.2 %, respectively. Other isolates belonged to 29 different serotypes, anyone of which was present in less than 5 %. *Salmonella* 1, 4, [5], 12:i:-, a monophasic variant of *S. typhimurium*, has increased considerably since the mid-1990s and currently represents one of the most common serotypes causing infection in many countries and Guangdong Province in China [21, 22]. As the third most prevalent serotype, *Salmonella* 1, 4, [5], 12:i:- highlighted the importance associated with childhood diarrheal disease in this study. Of the 37 *Shigella* isolates, *Shigella sonnei* was the most prevalent species, accounting for 86.5 %. Four *Shigella flexneri* isolates were represented by serotypes 2a and 4c, and only one *Shigella dysenteriae* strain was detected. The clear predominance of *S. sonnei* differs from that found in the studies of other provinces in China and many developing countries [6, 7, 12, 23]; however, several Asian countries have recently experienced a transition from *S. flexneri* to *S. sonnei*, probably due to improvement of overall nutritional status, sanitation and socioeconomic status [24–26]. The dominant serotype of *V. parahaemolyticus* was O3:K6, accounting for 80 %. Two types of K untypeable (KUT), namely, O1:KUT and O3:KUT, accounted for 10 %, respectively.

### Prevalence of pathogenic bacteria associated with era, season, age and clinical symptoms

**Fig. 1:**
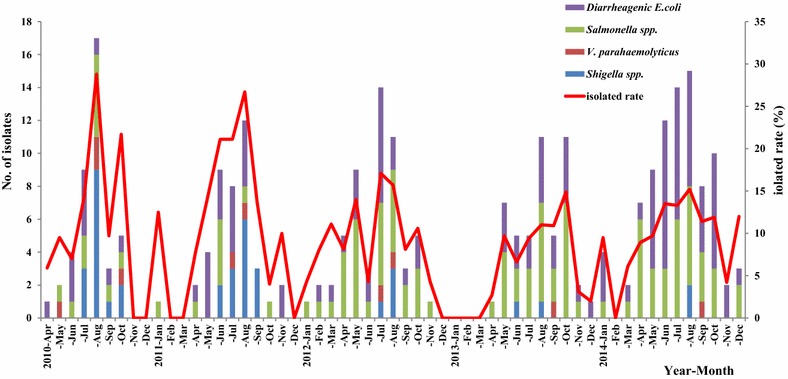
Isolated rate of enteropathogens per month in Beijing during 2010–2014

Over a period of five consecutive years, the overall isolation rate of enteric bacteria showed a gradual decrease from 15.1 % in 2010 to 10.7 % in 2014, with occasional interannual variation (Fig. 1). This isolation rate was much lower than the reported range of 28.0–55.1 % in other works [12, 27], taking into account the variation pertaining to geographical area, the types of enteric pathogens, diagnostic methods and social and educational conditions. In fact, recent studies have also shown lower incidence of infections in economically developed regions, such as Shanghai, in China [7, 28] because the progress of sanitary conditions has reduced the risk factors of diarrhea. In this study, different pathogens showed various change patterns over time. DEC was identified (with an isolation rate of 4.6 %) as the most prevalent pathogen, with an increased trend during the past 5 years. *Salmonella*, with an isolation rate of 4.3 %, has remained fairly constant in the past decade in China [5, 7, 21, 29, 30] but is much higher than in Bangladesh (0.78 %) [31] and lower than in Brazil (38.4 %) [9]. The present study also showed a significant decrease in diarrhea caused by *Shigella* (1.4 %) and *V. parahaemolyticus* (0.4 %). Although these results were consistent with multicenter data throughout China [6, 7], due to the limited data covering only a few years, it could not be established whether this trend will be maintained or vary in subsequent years. Further studies in the same area are necessary to definitively confirm these trends and to verify whether they truly correspond to an epidemiologic change or are just a response to exceptional situations.

The etiologic bacteria were isolated throughout the year, with a seasonal peak in summer (June–August) (Fig. 1; Table 1), which was also observed in other studies and is strongly associated with environmental factors [32], such as ambient temperature, precipitation and humidity. Beijing, with a high population density, is located in Northeastern China in the northern warm zone. In summer, the hot and humid climate facilitates microbial breeding, and food is more vulnerable to bacterial contamination, which may influence the survival and transmission of pathogens. This study showed that *Shigella*-caused diarrhea had a distinct peak in August. *V. parahaemolyticus* prevalence presented no remarkable seasonal trend because of the limited number of isolates. *Salmonella* and DEC were detected throughout the year with seasonal fluctuation.

The prevalence of pathogen categories was correlated with outpatient age (Table 1). The proportion of all causative bacteria was highest in children aged 3–5 years (19.1 %, *P* < 0.001). *Shigella* infection was the highest in the age group of 5 years (13.5 %, *P* < 0.001). *Salmonella*-caused diarrhea was most common at the age of 3–4 years (8.4 %, *P* < 0.001), whereas DEC infection in the group of 3–4 years (3.8 %) was secondary to the group of 1–2 years (6.9 %) (*P* = 0.002). *V. parahaemolyticus* showed no variation with age. Therefore, continuous surveillance targeting high-risk groups is needed to monitor the variation of etiology and epidemiological features.

The prevalence of clinical symptoms was also correlated with the distribution of pathogen categories (Table 1). Fever was the most common in all bacterial infections, except *V. parahaemolyticus* (*P* < 0.001). Abdominal pain and vomiting were the highest in *Shigella* infection (18.9 and 24.3 %, respectively, *P* < 0.001). *Salmonella* infection caused mucus stool, watery stool and bloody stool in 79.8, 18.3 and 6.4 % of cases, respectively (all *P* < 0.05), whereas *Shigella* infection was significantly associated with only bloody stool (8.1 %, *P* = 0.024).

### Antimicrobial resistance of *Salmonella* and *Shigella*

Antimicrobial drug testing was performed on 109 *Salmonella* isolates and 37 *Shigella* isolates (Table 3). For the *Salmonella* isolates, resistance to nalidixic acid (NAL) was most common (56.0 %), followed by resistance to sulfisoxazole (SUL) (55.0 %), ampicillin (AMP) (46.8 %), streptomycin (STR) (44.0 %), tetracycline (TET) (29.4 %) and chloramphenicol (CHL) (16.5 %). The resistance profiles differed among different serotypes. *S. typhimurium* and *Salmonella* 1, 4, [5], 12:i:- isolates showed significantly higher resistance to most antibiotics than *S. enteritidis* isolates, similarly to previous studies [6, 21, 33]. Overall, the resistance of other serotypes was relatively lower than that of the top three dominant serotypes. Approximately 50 % of *Salmonella* isolates were resistant to at least three antimicrobials, 41.3 % were resistant to four, 22.9 % were resistant to five and 12.8 % were resistant to six. Many different patterns of resistance were observed with two predominant resistance patterns: AMP-STR-SUL-TET (n = 7, 6.4 %) and AMP-STR-SUL-NAL (n = 6, 5.5 %).

**Table 3:** Antimicrobial resistance among *Salmonella* and *Shigella* isolates by serovars

Antimicrobial	No. (%) resistance of S*almonella species*						No. (%) resistance of *Shigella species*		
	*S. enteritidis* (n = 35)	*S. typhimurium* (n = 15)	1, 4, [5], 12:i:- (n = 13)	*S. agona* (n = 10)	Overall (n = 109)	*S. sonnei* (n = 32)	*S. flexneri* (n = 4)	*S. dysenteriae* (n = 1)	Overall (n = 37)
AMP	21 (60.0)	12 (80.0)	11 (84.6)	1 (10.0)	51 (46.8)	31 (96.9)	4 (100.0)	0 (0.0)	35 (94.6)
CHL	3 (8.6)	8 (53.3)	4 (30.8)	1 (10.0)	18 (16.5)	2 (6.3)	4 (100.0)	0 (0.0)	6 (16.2)
CIP	1 (2.9)	6 (40.0)	3 (23.1)	0 (0.0)	10 (9.2)	0 (0.0)	1 (25.0)	0 (0.0)	1 (2.7)
CRO	7 (20.0)	0 (0.0)	2 (15.4)	0 (0.0)	10 (9.2)	10 (31.3)	1 (25.0)	0 (0.0)	11 (29.7)
GEN	3 (8.6)	7 (46.7)	3 (23.1)	1 (10.0)	14 (12.8)	30 (93.8)	0 (0.0)	0 (0.0)	30 (81.1)
NAL	34 (97.1)	9 (60.0)	5 (38.5)	1 (10.0)	61 (56.0)	32 (100.0)	4 (100.0)	0 (0.0)	36 (97.3)
STR	15 (42.9)	10 (66.7)	12 (92.3)	2 (20.0)	48 (44.0)	32 (100.0)	4 (100.0)	1 (100.0)	37 (100.0)
SUL	19 (54.3)	12 (80.0)	9 (69.2)	2 (20.0)	60 (55.0)	32 (100.0)	1 (25.0)	1 (100.0)	34 (91.9)
TET	7 (20.0)	10 (66.7)	10 (76.9)	1 (10.0)	32 (29.4)	12 (37.5)	4 (100.0)	1 (100.0)	17 (45.9)
Multidrug-resistance (MDR)									
MDR3	22 (62.9)	12 (80.0)	11 (84.6)	1 (10.0)	54 (50.0)	32 (100)	4 (100.0)	1 (100)	37 (100)
MDR4	17 (48.6)	12 (80.0)	9 (69.2)	1 (10.0)	45 (41.3)	31 (96.9)	4 (100.0)	0 (0.0)	35 (94.6)
MDR5	7 (20.0)	10 (66.7)	5 (38.5)	1 (10.0)	25 (22.9)	31 (96.9)	4 (100.0)	0 (0.0)	35 (94.6)
MDR6	2 (5.7)	7 (46.7)	4 (30.8)	1 (10.0)	14 (12.8)	18 (56.3)	3 (75.0)	0 (0.0)	21 (56.8)

For 109 *Salmonella* isolates, two dominant resistance patterns are AMP-STR-SUL-TET (n = 7, 6.4 %) and AMP-STR-SUL-NAL (n = 6, 5.5 %)

For 37 *Shigella* isolates, two dominant resistant patternsare AMP-GEN-NAL-STR-SUL (n = 12, 32.4 %) and AMP-GEN-NAL-STR-SUL-TET (n = 8, 21.6 %)

AMP ampicillin, CRO ceftriaxone, CIP ciprofloxacin, NAL nalidixic acid, TET tetracycline, GEN gentamicin, CHL chloramphenicol, SUL sulfisoxazole, STR streptomycin, MDR3 resistance to any three antibiotics, MDR4 resistance to any four antibiotics and so on

The resistance of *Shigella* to nearly all antimicrobials was much more obvious than *Salmonella*, except CHL and ciprofloxacin (CIP). Of the 37 *Shigella* isolates, resistance to STR was most common (100 %), followed by resistance to NAL (97.3 %), AMP (94.6 %), SUL (91.9 %) and gentamicin (GEN) (81.1 %). Moderate resistance to TET (45.9 %) and ceftriaxone (CRO) (29.7 %) was also observed; however, resistance to CHL and CIP was relatively low (16.2 and 2.7 %, respectively). All *Shigella* isolates were resistant to at least three antimicrobials, 94.6 % of isolates were resistant to four or five antimicrobials and 56.8 % were resistant to six. The two dominant antimicrobial resistant profiles were AMP-GEN-NAL-STR-SUL (n = 12, 32.4 %) and AMP-GEN-NAL-STR-SUL-TET (n = 8, 21.6 %). *S. sonnei* isolates were resistant to five (96.9 %) and six (56.3 %) antimicrobials, and of the four *S. flexneri* isolates, 100 and 75 %, respectively, were resistant to five and six antimicrobials. Although a similar pattern of resistance was reported in China and other Asian countries [6, 34, 35], the frequency of resistance reported here is higher, and the trend of multidrug resistance is more pronounced.

According to the WHO guidelines for the treatment of diarrhea, antimicrobials should not be used routinely, particularly for unknown causative agents. Antimicrobials are reliably helpful only for children with bloody diarrhea (probable shigellosis), suspected cholera with severe dehydration and serious non-intestinal infections, such as pneumonia [36]. Because the proportion of isolates resistant to AMP, CHL, SUL, STR, GEN and TET has increased substantially among *Shigella* isolates worldwide in the past decade, these drugs are no longer recommended as empirical therapy for shigellosis by the WHO. Cephalosporins and fluoroquinolones are two popular acceptable options to treat severe gastrointestinal infections caused by pathogenic bacteria. As the third generation of the above two kinds of antibiotics, CRO and CIP are the drugs of choice for all patients with bloody diarrhea according to the WHO, irrespective age [36]. However, the 9.3 and 29.7 % prevalence of resistance to CRO in *Salmonella* and *Shigella* isolates from diarrheal children was noteworthy in Beijing. Additionally, our study showed, respectively, 9.2 and 2.7 % resistance rates to CIP in *Salmonella* and *Shigella* isolates, which is an alarming development that may also exist in other regions, such as Shanghai and Anhui in China [37, 38].

Antibiotic use outside hospitals without a medical prescription is very common, and most patients (65 %) with a diarrheal illness in China are treated with antibiotics without identification of the causative pathogen [39]. In this study, 20.1 % of children had taken antibiotics prior to admission, whereas only 2.5 % had bloody diarrhea. The widespread use of antimicrobial agents in the treatment of infections is responsible for the alarming rate of increase of multiple antimicrobial resistance and poses a great challenge. Serious constraints should be imposed on the antibiotics available for treatment of diarrhea among pediatricians. Even for therapy, antimicrobial treatment strategies must be designed to ensure safety and efficacy based on current resistance patterns.

## Conclusion

The current surveillance system can identify only the common bacterial pathogens without understanding the patterns of viral and parasitic infection, which should be included to make the findings of the study more complete and valuable. To date, we can characterize only the resistance patterns for *Salmonella* and *Shigella*. As the active surveillance continues, we will further explore the etiologic spectrum and variation trends in antimicrobial resistance so that we can execute earlier and proper intervention to decrease the financial load on the patient and on health care.

## Methods

### Study design and population

The active surveillance program, the Enterobacterial Communicable Disease Surveillance (ECDS), was conducted from April 2010 to December 2014 in Beijing, the capital of China. Seventeen sentinel hospitals affiliated with six districts with different geographical and socioeconomic situation enrolled outpatients with acute diarrhea on two random days per week. A total of 2524 children under 5 years of age who were admitted with acute diarrhea diseases were included in this study. Clinical examination of each patient by a physician was followed by treatment and monitoring. The clinical and epidemiological information was recorded through the electronic gastrointestinal clinic surveillance and reporting system. Enrollment was subject to obtaining informed verbal consent from the parent or guardian who accompanied the child. All specimens were collected on the day of presentation by rectal swabs in Cary-Blair transport media and were immediately transported to the laboratory of Disease Prevention and Control (CDC) for processing within 24 h.

### Detection of bacteria

All specimens were processed by routine microbiologic and biochemical tests to identify different enteropathogenic bacteria. Each stool specimen was cultured by streaking on xylose-lysine deoxycholate (XLD) agar and *Salmonella*-*Shigella* agar (Becton–Dickinson Co., Sparks, MD, USA) for *Shigella* spp., and *Salmonella* spp., respectively, and incubating at 37 °C for 16–24 h. For *S. typhi*, swabs were enriched in Selenite brilliant-green broth (Becton–Dickinson Co., Sparks, MD, USA) at 37 °C overnight, followed by sub-culturing on the media described above. For selective enrichment of *Vibrio* spp., swabs were inoculated on peptone water containing 3 % NaCl, pH 8, were incubated at 37 °C overnight, inoculated on CHROMagar *Vibrio* media (CHROMagar Co., Paris, France), and incubated for 16–24 h. After culturing, at least five colonies morphologically resembling specific species were selected for further identification by biochemical testing according to standard methods. For other enteropathogenic bacteria, the systematic identification was confirmed with a VITEK 2 Compact instrument (bioMérieux, Marcyl’Etoile, France). Finally, serotyping was performed by slide agglutination test using commercially available antisera (*Salmonella* antisera from State Serum SSI Diagnostica, Denmark, *Shigella* and *Vibrio* antisera from Denka Seiken, Tokyo, Japan).

### Real-time PCR assay for diarrheagenic *E. coli*

Pathotypes of DEC were identified by real-time PCR, including EAEC, EIEC, EPEC, ETEC and STEC. *E. coli*-like and other gram-negative colonies growing on MacConkey agar were tested for the presence of virulence genes using seven sets of primers targeting *agg*R, *stx*1, *stx*2, *ipa*H, *bfp*A, *eae*, *elt* and *est* (Additional file 1). The real-time PCR assay was performed using the TaqMan^®^ Universal PCR Master Mix Kit (Applied Biosystems, USA) on an Applied Biosystems 7500 Real-Time PCR System. The reaction mixture consisted of 2X PCR Master Mix, 200 nM of forward and reverse primers, and 200 nM of TaqMan probe. The amplification profile consisted of heat activation at 95 °C for 10 min; 40 cycles of denaturation at 95 °C for 15 s, annealing, extension, and fluorogenic signal detection at 60 °C for 1 min. The assay was considered positive when the number of cycles to detection was 35 or less with the standard S curve.

### Antimicrobial susceptibility testing

The antibiotic resistance of the bacterial isolates was tested using the Kirby-Bauer disk diffusion method following the guidelines of the Clinical and Laboratory Standards Institute (CLSI 2010). *E. coli* (ATCC 25922) was included in the test for quality control. The disk concentrations of 9 antimicrobial agents (Oxide, UK) were as follows: 10 μg AMP, 30 μg CRO, 5 μg CIP, 30 μg NAL, 30 μg TET, 10 μg GEN, 30 μg CHL, 300 μg SUL, and 10 μg STR. Multidrug resistance was defined as resistance to at least three classes of antibiotics.

### Statistical analysis

All data were entered in an Excel database (Additional file 2) (Microsoft Corporation, USA). Statistical analysis was performed with SPSS/11.5 software (SPSS Inc., USA). Comparison of proportions and statistical significance were calculated using a two-tailed Chi square test or Fisher’s exact test with a *P* < 0.05 considered significant.

## Additional files

10.1186/s13099-016-0116-2 Primers and probes used for real-time PCR for detection of diarrhoeagenic E. coli.
10.1186/s13099-016-0116-2 Basic information and antimicrobial resistance of all strains isolated from diarrheal children.

## Abbrevations

DEC: diarrheagenic *Escherichia coli*
EPEC: enteropathogenic *Escherichia coli*
EAEC: enteroaggregative *Escherichia coli*
ETEC: enterotoxigenic *Escherichia coli*
EIEC: enteroinvasive *Escherichia coli*
STEC: shiga toxin-producing *Escherichia coli*
KUT: Kuntypeable
NAL: nalidixic acid
SUL: sulfisoxazole
AMP: ampicillin
STR: streptomycin
TET: tetracycline
CHL: chloramphenicol
CIP: ciprofloxacin
GEN: gentamicin
CRO: ceftriaxone
MDR: multidrug-resistance
ECDS: enterobacterial communicable disease surveillance
CDC: disease prevention and control
XLD: xylose-lysine deoxycholate
CLSI: Clinical and Laboratory Standards Institute
