# Supplementary material for: Prevalence and antibiotic resistance of bacterial pathogens isolated from childhood diarrhea in Beijing, China (2010–2014)
Source: Gut Pathog. 2016 Jun 13;8:31. doi: 10.1186/s13099-016-0116-2 (PMC4906916; doi:10.1186/s13099-016-0116-2)
Supplement: Supplementary file 1 — 10.1186/s13099-016-0116-2 Primers and probes used for real-time PCR for detection of diarrhoeagenic E. coli. [file 13099_2016_116_MOESM1_ESM.doc]

**Primers and probes used for real-time PCR for detection of diarrhoeagenic *E.coli***

|  | Target | Primer and probe | Sequence（5’-3’） |
| --- | --- | --- | --- |
| EHEC | *stx*1 | stx1-F | CATCGCGAGTTGCCAGAAT |
|  |  | stx1-R | TGCGTAATCCCACGGACTCT |
|  |  | stx1-Probe | FAM-TGCCGGACACATAGAAGGAAACTCATCA-TAMARA |
|  | *stx*2 | stx2-F | GCTGGAATCTGCAACCGTTACT |
|  |  | stx2-R | CACGAATCAGGTTATGCCTCAGT |
|  |  | stx2-Probe | FAM-CTGCACTTCAGCAAATCCGGAGCCT-TAMARA |
| EPEC | *eae* | eae-F | CCGATTCCTCTGGTGACGA |
|  |  | eae-R | CCACGGTTTATCAAACTGATAACG |
|  |  | eae-Probe | FAM-CGTCATGGTACGGGTAA-TAMARA |
|  | *bfp*A | bfpA-F | TCTTTGATTGAATCGGCAATG |
|  |  | bfpA-R | TGTGACTTATTGGAATCAGACGC |
|  |  | bfpA-Probe | FAM-TGCGCTTGCTGCAACCGTTACTGC-TAMARA |
| ETEC | *elt* | elt-F | GTTGACTGCCCGGGACTTC |
|  |  | elt-R | CGGAATATCGCAACACACAAAT |
|  |  | elt-Probe | FAM-CCTGAAATGTTGCGCCGCTCTTAAATG-TAMARA |
|  | *est* | est-F | ACAGACATCATCAGAATCAGAACAAAT |
|  |  | est-R | AGTGGTCCTGAAAGCATGAATAGTAG |
|  |  | est-Probe | FAM-CACCCGGTACAAGCAGGATTACAACACA-TAMARA |
| EAEC | *agg*R | aggR-F | CAGAATCGTCAGCATCAGCTACA |
|  |  | aggR-R | AAGGATGCCCTGATGATAATATACG |
|  |  | aggR-Probe | FAM-ACCAATTCGGACAACTGCAAGCATCT-TAMARA |
| EIEC | *ipa*H | ipaH-F | CGTGAACAGGTCGCTGCAT |
|  |  | ipaH-R | CAGCAGCAACAGCGAAAGACT |
|  |  | ipaH-Probe | FAM-TCAGTGCCTCTGCGGAGCTTCGAC-TAMARA |
